# Comparative Effectiveness of Switching to Bictegravir From Dolutegravir-, Efavirenz-, or Raltegravir-Based Antiretroviral Therapy Among Individuals With HIV Who are Virologically Suppressed

**DOI:** 10.1093/ofid/ofae446

**Published:** 2024-08-07

**Authors:** Isaac Núñez, Yanink Caro-Vega, Conor MacDonald, Juan Luis Mosqueda-Gómez, Alicia Piñeirúa-Menéndez, Anthony A Matthews

**Affiliations:** Department of Medical Education, Instituto Nacional de Ciencias Médicas y Nutrición Salvador Zubirán, Mexico City, Mexico; Division of Postgraduate Studies, Faculty of Medicine, Universidad Nacional Autónoma de México, Mexico City, Mexico; Unit of Epidemiology, Institute of Environmental Medicine, Karolinska Institutet, Stockholm, Sweden; Department of Infectious Diseases, Instituto Nacional de Ciencias Médicas y Nutrición Salvador Zubirán, Mexico City, Mexico; Unit of Epidemiology, Institute of Environmental Medicine, Karolinska Institutet, Stockholm, Sweden; High Specialty Regional Hospital Bajio, Health Secretariat, León, Mexico; Consorcio de Investigación en Salud, Cuernavaca, Mexico; Unit of Epidemiology, Institute of Environmental Medicine, Karolinska Institutet, Stockholm, Sweden

**Keywords:** bictegravir, health policy, HIV, target trial emulation, causal inference

## Abstract

**Background:**

We aimed to determine the effectiveness of switching to bictegravir in maintaining an undetectable viral load (<50 copies/mL) among people with HIV (PWH) as compared with continuing dolutegravir-, efavirenz-, or raltegravir-based antiretroviral therapy using nationwide observational data from Mexico.

**Methods:**

We emulated 3 target trials comparing switching to bictegravir vs continuing with dolutegravir, efavirenz, or raltegravir. Eligibility criteria were PWH aged ≥16 years with a viral load <50 copies/mL and at least 3 months of current antiretroviral therapy (dolutegravir, efavirenz, or raltegravir) between July 2019 and September 2021. Weekly target trials were emulated during the study period, and individuals were included in every emulation if they continued to be eligible. The main outcome was the probability of an undetectable viral load at 3 months, which was estimated via an adjusted logistic regression model. Estimated probabilities were compared via differences, and 95% CIs were calculated via bootstrap. Outcomes were also ascertained at 12 months, and sensitivity analyses were performed to test our analytic choices.

**Results:**

We analyzed data from 3 028 619 PWH (63 581 unique individuals). The probability of an undetectable viral load at 3 months was 2.9% (95% CI, 1.9%–3.8%), 1.3% (95% CI, .9%–1.6%), and 1.2% (95% CI, .8%–1.7%) higher when switching to bictegravir vs continuing with dolutegravir, efavirenz, and raltegravir, respectively. Similar results were observed at 12 months and in other sensitivity analyses.

**Conclusions:**

Our findings suggest that switching to bictegravir could be more effective in maintaining viral suppression than continuing with dolutegravir, efavirenz, or raltegravir.

## Teaser

Among individuals with HIV and a suppressed viral load, switching to bictegravir-based antiretroviral therapy could be more effective in maintaining viral suppression than continuing dolutegravir-, efavirenz-, or raltegravir-based therapy.

Bictegravir and dolutegravir are currently recommended in combination with 2 nucleoside reverse transcriptase inhibitors as the preferred initial antiretroviral therapy in people with HIV [1, 2]. Guidelines also support switching to these antiretrovirals in those who are currently taking another treatment (eg, nonnucleoside reverse transcriptase inhibitors) and have an undetectable viral load [2]. Changing antiretroviral therapy can be useful to simplify treatment (ie, by taking fewer pills), reduce side effects, and ensure that health systems provide the best currently available treatment [3].

Homogenizing antiretroviral therapy via drug rollouts allows health care systems and governments to purchase a large amount of a particular antiretroviral in bulk, which grants leveraging power to reduce costs [4]. Such a strategy was implemented by Mexico, and the “consolidated purchase” of bictegravir took place in 2019 [5, 6]. Consequently, numerous individuals taking dolutegravir-, efavirenz-, or raltegravir-based therapy switched to bictegravir. There are currently about 246 000 people in Mexico with HIV, many of whom could potentially benefit from this strategy [7].

There are several caveats with this strategy. First, only 1 trial has shown that switching from dolutegravir to bictegravir is noninferior to continuing dolutegravir, and its limited sample size does not allow to determine if 1 strategy is comparatively more effective, especially if the true difference is small [8]. As the intention is to switch many individuals to bictegravir on a national level, small differences in effectiveness become relevant when deciding which integrase inhibitor to buy for a whole country. Furthermore, no evidence exists on the comparative effectiveness of switching from efavirenz or raltegravir, as commonly prescribed in Mexico, to bictegravir. Given their lower cost (in particular, the availability of generic efavirenz), similar effectiveness might discourage the universal switch to bictegravir in those who are virally suppressed [9].

Here, we specify the protocol of 3 hypothetical target trials that would compare switching to bictegravir from dolutegravir-, efavirenz-, and raltegravir-based antiretroviral therapy regimens with continuing the same treatment among people with HIV who have an undetectable viral load. We then emulated the target trials using observational data from Mexico.

## METHODS

### Study Design

Causal inference from observational data can be considered an endeavor to emulate a pragmatic randomized trial—the target trial—that would answer the research question of interest [10, 11]. Emulating a target trial has 2 steps: (1) specify the study protocol of the target trial and (2) emulate the target trial using the available observational data. In this section, we first specify the 3 target trials that would answer our research questions, including eligibility criteria, treatment strategies, treatment assignment, outcomes, follow-up, causal contrasts, and statistical analysis. Then, we explain how we emulated them using observational data from Mexico, focusing on the protocol modifications required to accommodate the available data.

### Target Trials

Table 1 summarizes the key components of the 3 target trial protocols. We designed the target trials analogous to published randomized trials of antiretroviral therapy effectiveness [8].

**Table 1.** Protocol of 3 Target Trials and Their Emulations to Estimate the Comparative Effectiveness of Switching to Bictegravir vs Continuing With Dolutegravir, Efavirenz, or Raltegravir in Mexico, 2019–2021

Protocol Component	Target Trials	Target Trial Emulation With SALVAR
Eligibility criteria	Age ≥16 y at moment of eligibility Viral load <50 copies/mL No diagnosis of tuberculosis at moment of eligibility for antiretroviral switch No pregnancy at moment of eligibility for antiretroviral switch	Same as target trials except excluded individuals with double-dose dolutegravir in target trial 1
Target trial 1^a^	Undergoing dolutegravir-based ART during the week of the target trial and for at least the last 3 mo No 2-drug regime	
Target trial 2^b^	Undergoing efavirenz-based ART during the week of the target trial and for at least the last 3 mo	
Target trial 3^c^	Undergoing raltegravir based-ART during the week of the target trial and for at least the last 3 mo	
Treatment strategies	Assigned to switching bictegravir-based ART (50 mg daily), only single-tablet regime coformulated with emtricitabine (200 mg daily), and tenofovir alafenamide (25 mg daily)	Same as target trial; assignment was determined with data from SALVAR
Target trial 1^a^	Continuing on dolutegravir-based ART (50 mg daily; single- or multitablet regime) Could be administered with (1) tenofovir alafenamide (25 mg daily) or tenofovir disoproxil fumarate (300 mg daily) or abacavir (600 mg daily) and (2) emtricitabine (200 mg daily) or lamivudine (300 mg daily)	
Target trial 2^b^	Continuing on efavirenz-based ART (600 mg daily; single- or multitablet regime) Could be administered with (1) tenofovir alafenamide (25 mg daily) or tenofovir disoproxil fumarate (300 mg daily) or abacavir (600 mg daily) and (2) emtricitabine (200 mg daily) or lamivudine (300 mg daily)	
Target trial 3^c^	Continuing on raltegravir-based ART (400 mg twice daily; only multitablet regime) Could be administered with (1) tenofovir alafenamide (25 mg daily) or tenofovir disoproxil fumarate (300 mg daily) or abacavir (600 mg daily) and (2) emtricitabine (200 mg daily) or lamivudine (300 mg daily)	
Treatment assignment	Random open-label assignment with no stratification: bictegravir switch or dolutegravir, efavirenz, or raltegravir maintenance	Treatment assignment is randomized conditional on the following covariates: age, gender, year of ART start, baseline CD4 cell count, state, prison status
Outcomes	Risk of undetectable viral load (<50 copies/mL) at 3 mo within ±8 wk	Same as target trial
Follow-up	Started at baseline and ended at evaluation of outcome	Same as target trial
Causal contrast	Intention-to-treat effect	Observational analogue of intention-to-treat effect
Statistical analysis	Intention-to-treat analysis Logistic regression model for the outcome with an indicator for the assigned arm and adjusted for prognostic factors unbalanced among arms Estimated probabilities from the model standardized to the distribution of the baseline covariates in each arm Probabilities compared via differences	Same as target trial with inclusion of all covariates in model

Abbreviations: ART, antiretroviral therapy; SALVAR, System of Administration, Logistics, and Vigilance of Antiretrovirals.

^a^Target trial 1: switching to bictegravir vs continuing dolutegravir.

^b^Target trial 2: switching to bictegravir vs continuing efavirenz.

^c^Target trial 3: switching to bictegravir vs continuing raltegravir.

The eligibility criteria would be people with HIV who were aged ≥16 years between July 2019 and September 2021; who were currently using dolutegravir, efavirenz, or raltegravir (for each of the 3 target trials) and had been so for at least 3 months; who had not used bictegravir before; who had at least 1 viral load measured within the last year and the most recent one was undetectable (defined as <50 copies/mL); and who did not have tuberculosis and were not pregnant. Eligible individuals would be randomly assigned to switching to an antiretroviral therapy regimen based on bictegravir (50 mg once daily) or continuing their current therapy (dolutegravir, efavirenz, or raltegravir). Bictegravir would be administered as a single-tablet regime in combination with emtricitabine (200 mg daily) and tenofovir alafenamide (25 mg daily). Dolutegravir, efavirenz, and raltegravir could be combined with (1) tenofovir alafenamide (25 mg daily) or tenofovir disoproxil fumarate (300 mg daily) or abacavir (600 mg daily) and (2) emtricitabine (200 mg daily) or lamivudine (300 mg daily). Dolutegravir and efavirenz could come as single- or multitablet regimes, with raltegravir only as a multitablet regime. The primary outcome would be an undetectable viral load at 3 months to ascertain early cases of virologic failure, and the secondary outcome would be undetectable viral load at 12 months (similar to the Food and Drug Administration snapshot algorithm) [12]. The causal contrast of interest would be the intention-to-treat effect.

In each of the 3 target trials, the intention-to-treat analysis would use a logistic regression model for the outcome with an indicator for the assigned arm; prognostic factors unbalanced among arms would be included as adjustment variables. Estimated probabilities from the model would be standardized to the distribution of the baseline covariates in each arm. Probabilities would then be compared via differences. Nonparametric bootstrapping with 500 samples would be used to calculate 95% CIs.

### Observational Emulation of the Target Trials

We used observational data from the registry of the System of Administration, Logistics, and Vigilance of Antiretrovirals (SALVAR, for its initials in Spanish) to emulate the target trials previously specified. SALVAR started operating at a national level in 2008. Its initial function was to administer the purchase of antiretrovirals. Currently, SALVAR oversees the government acquisition of antiretrovirals and their delivery to all uninsured people with HIV in the country (all of whom fall under coverage by the health secretariat). SALVAR is estimated to collect data on about half the people with HIV in Mexico. During each health care contact that a patient has with a provider, clinical data such as CD4 cell count, HIV viral load, and date of antiretroviral prescription are collected. Mortality is routinely ascertained by matching the database with the Mexican death registry. Each component of the target trial protocol was emulated as closely as possible with these data.

The eligibility criteria were the same as the target trials. However, the presence of tuberculosis was operationalized as the receipt of a modified antiretroviral dose regimen compatible with such a diagnosis (ie, double-dose dolutegravir for the “switching to bictegravir vs continuing dolutegravir” target trial emulation). As data collected on pregnancy do not have specific dates, persons reported as pregnant at any time were excluded from the main analysis. Those without data on baseline confounders or with unlikely values of CD4 cell counts (>3000 cells/mm^3^) were also excluded.

Treatment strategies were the same as the target trials. Due to the nature of the eligibility criteria, people could be eligible at several times. Thus, from the third week of July 2019 (when the first individuals switched to bictegravir) until the last week of September 2021 (113 weeks), we emulated each of the 3 target trials every week: 1 for switching to bictegravir vs continuing dolutegravir, 1 for switching to bictegravir vs continuing efavirenz, and 1 for switching to bictegravir vs continuing raltegravir. If individuals met the eligibility criteria during a given week, they were included in that week's target trial emulation. If they switched to bictegravir during that week, they were then assigned to the switch strategy; otherwise, they were assigned to the strategy of continuing their current antiretroviral (dolutegravir, efavirenz, or raltegravir). Individuals could participate in more than 1 target trial emulation if they continued to meet eligibility criteria in subsequent weeks.

We assumed that assignment was random within levels of the following baseline covariates: CD4 cell count (cells/mm^3^; linear and quadratic), living in a prison (yes/no), state of residence, calendar year of the trial (2019, 2020, or 2021), gender (cis man, cis woman, trans man, or trans woman), and age (linear and quadratic). These covariates were selected due to their possible causal association with the exposure, the outcome, or both [13].

As the observational data capture viral load only when individuals attend a clinic, the outcome was defined as a viral load measure taken at 3 or 12 months ± 8 weeks following treatment assignment. If more than 1 viral load measure was available during this period, the later one was used. Given that the last data available from SALVAR were from December 2021, for the outcome measured at 12 months, we emulated only those target trials that would allow an outcome to be ascertained at 12 months (83 total weekly target trials). Due to the observational nature of the data, individuals may not have a viral load measured within the stipulated period. The management of these missing data is discussed below.

The intention-to-treat analysis was the same as for the target trials, with all baseline covariates adjusted for. For each of the 3 target trials, we combined the data from all weekly target trials into a single data set and introduced a variable for the trial number in which that person participated. This trial number was also included as a covariate in the regression model. As outcomes were captured only for those who had their viral loads measured within the predefined window (ie, 3 months ± 8 weeks and 12 months ± 8 weeks), the logistic regression model was fit within the individuals with nonmissing outcome data; then, this model was used to standardize to the distribution of baseline covariates of all eligible individuals. This approach leads to an unbiased effect estimate under the assumption that outcome data are missing at random, conditional on covariates included in the model [14–16]. Additionally, to estimate the total effect, persons who died were not censored [17]. Nonparametric bootstrapping with 500 samples was used to calculate 95% CIs to avoid relying on parametric assumptions [18]. All analyses were performed with R version 4.1.2.

### Sensitivity Analyses

We assessed how robust our effect estimates were to several assumptions and analytic choices. Specifically, we aimed to understand the impact of:

- Our modeling assumptions by performing an inverse probability weighted analysis
- Our missing data assumption by standardizing estimates to the distribution of baseline covariates of only those with complete outcome data
- Extending the outcome detection period to 3 months ± 10 weeks and 12 months ± 12 weeks
- Changing the definition of undetectable viral load to a more lenient value by increasing it to <200 copies/mL as an inclusion criterion and as the outcome
- Considering the possibility of excluding individuals incorrectly classified as pregnant by dropping the “no pregnancy” exclusion criterion.

Further details regarding the sensitivity analyses are provided in the Supplementary material.

### Patient Consent

This study was approved by the ethics committee of the Instituto Nacional de Ciencias Médicas y Nutrición Salvador Zubirán (ref 4349). No informed consent was required due to the use of routinely collected data.

## RESULTS

### Switching to Bictegravir vs Continuing Dolutegravir

**Figure 1.**
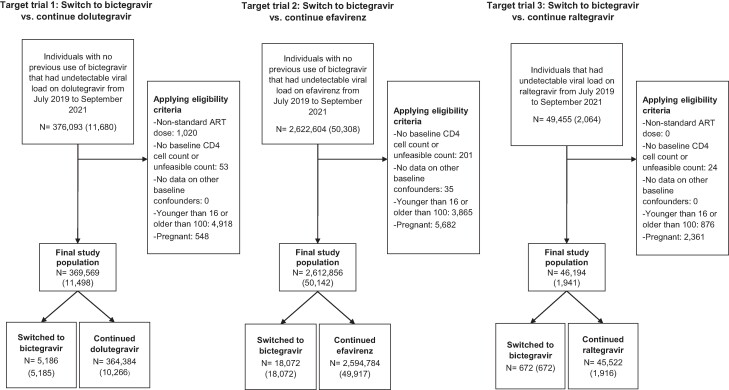
Flowchart for eligibility for 3 target trial emulations of switching to bictegravir vs continuing dolutegravir, efavirenz, or raltegravir therapy among people with HIV in Mexico who are virologically suppressed, 2019–2021. Number of unique individuals are shown in parentheses. ART, antiretroviral therapy.

Overall, 369 569 people with HIV were eligible for the target trial emulation that involved switching to bictegravir vs continuing dolutegravir (flowchart in Figure 1), which consisted of 5186 unique individuals in the bictegravir arm and 10 266 in the dolutegravir arm. Median baseline CD4 cell count was similar between study arms (564 cells/mm^3^ in the bictegravir arm vs 526 cells/mm^3^ in the dolutegravir arm), and most individuals were cis men. Full characteristics of included individuals are shown in Table 2, and state of residence is shown in Supplementary Table 1. The percentage who died before the main outcome period (3 months) was lower in those who received bictegravir than those who continued dolutegravir (0.01% vs 0.07%). Outcome data were available for 27.6% in the dolutegravir arm and 39.2% in the bictegravir arm.

**Table 2.** Baseline Characteristics for 3 Target Trial Emulations of Switching to Bictegravir vs Continuing With Dolutegravir, Efavirenz, or Raltegravir Among People With HIV in Mexico Who Were Virologically Suppressed, 2019–2021

	Target Trial 1^a^		Target Trial 2^b^		Target Trial 3^c^	
	Bictegravir (n = 5185)	Dolutegravir (n = 364 384)	Bictegravir (n = 18 072)	Efavirenz (n = 2 594 784)	Bictegravir (n = 672)	Raltegravir (n = 45 522)
Unique individuals	5185	10 266	18 072	49 917	672	1916
Year of target trial						
2019	4484 (86.5)	68 564 (18.8)	6546 (36.2)	621 666 (24.0)	297 (44.2)	9178 (20.2)
2020	438 (8.4)	170 038 (46.7)	5714 (31.6)	1 358 863 (52.4)	193 (28.7)	20 866 (45.8)
2021	263 (5.1)	125 782 (34.5)	5812 (32.2)	614 255 (23.7)	182 (27.1)	15 478 (34.0)
Gender						
Cis man	4060 (78.3)	294 753 (80.9)	14 634 (81.0)	2 084 081 (80.3)	209 (31.1)	15 078 (33.1)
Cis woman	1018 (19.6)	62 374 (17.1)	3064 (17.0)	459 205 (17.7)	459 (68.3)	30 093 (66.1)
Trans woman	107 (2.1)	7257 (2.0)	374 (2.1)	51 498 (2.0)	4 (0.6)	351 (0.8)
Age, y	40 (32–50)	39 (31–49)	39 (31–49)	39 (32–48)	31 (26–40)	33 (26–41)
Baseline CD4 cell count, cells/mm^3^	564 (380–778)	526 (338–752)	551 (379–760)	545 (374–748)	508 (340–727)	539 (346–772)
Imprisoned						
Yes	45 (0.9)	4288 (1.2)	151 (0.8)	31 220 (1.2)	3 (0.4)	447 (1.0)
No	5140 (99.1)	360 096 (98.8)	17 921 (99.2)	2 563 564 (98.8)	669 (99.6)	45 075 (99.0)

Data are presented as No. (%) or median (IQR).

^a^Target trial 1: switching to bictegravir vs continuing dolutegravir.

^b^Target trial 2: switching to bictegravir vs continuing efavirenz.

^c^Target trial 3: switching to bictegravir vs continuing raltegravir.

The adjusted probability of individuals with a viral load <50 copies/mL at 3 months was 96.0% (95% CI, 95.1%–96.8%) in those who switched to bictegravir and 93.1% (95% CI, 92.9%–93.3%) in those who continued dolutegravir, which resulted in a difference of 2.9% (95% CI, 1.9%–3.8%). The results were similar when evaluated at 12 months (Table 3).

**Table 3.** Adjusted Probabilities and Differences of Undetectable Viral Load at 3 and 12 Months After Switching to Bictegravir vs Continuing With Dolutegravir, Efavirenz, or Raltegravir Among People With HIV in Mexico Who Were Virologically Suppressed, 2019–2021

	Target Trial 1^a^		Target Trial 2^b^		Target Trial 3^c^	
Viral Load <50 Copies/mL	Adjusted Probability	Difference	Adjusted Probability	Difference	Adjusted Probability	Difference
At 3 mo						
Switch to bictegravir	96.0 (95.0–96.8)	2.9 (1.9–3.8)	97.0 (96.5–97.3)	1.3 (.9–1.6)	96.9 (96.5–97.3)	1.2 (.8–1.7)
Continue previous treatment	93.1 (92.9–93.3)	…	95.7 (95.6–95.7)	…	95.7 (95.1–96.3)	…
At 12 mo						
Switch to bictegravir	94.3 (92.9–95.4)	1.4 (.0–2.5)	95.8 (95.3–96.3)	1.2 (.6–1.7)	95.7 (95.1–96.3)	1.1 (.5–1.7)
Continue previous treatment	92.9 (92.7–93.1)	…	94.7 (94.6–94.7)	…	94.6 (94.4–94.7)	…

Data are presented as percentage (95% CI). We adjusted for the following baseline covariables: CD4 cell count, living in a prison, state of residence, year of target trial, gender, and age.

^a^Target trial 1: switching to bictegravir vs continuing dolutegravir.

^b^Target trial 2: switching to bictegravir vs continuing efavirenz.

^c^Target trial 3: switching to bictegravir vs continuing raltegravir.

### Switching to Bictegravir vs Continuing Efavirenz

Overall, 2 612 856 people with HIV were eligible for the target trial emulation that involved switching to bictegravir vs continuing efavirenz (flowchart in Figure 1), which consisted of 18 072 unique individuals in the bictegravir arm and 49 917 in the efavirenz arm. Median baseline CD4 cell count was similar between study arms (551 cells/mm^3^ in the bictegravir arm vs 545 cells/mm^3^ in the efavirenz arm), and most individuals were cis men. Full characteristics of included individuals are shown in Table 2, and state of residence is shown in Supplementary Table 1. The same percentage died before the main outcome period (3 months) in both arms (0.04%). Outcome data were available for 28.4% in the efavirenz arm and 37.5% in the bictegravir arm.

The adjusted probability of having a viral load <50 copies/mL at 3 months was 97.0% (95% CI, 96.5%–97.3%) in those who switched to bictegravir and 95.7% (95% CI, 95.6%–95.7%) in those who continued efavirenz, which resulted in a difference of 1.3% (95% CI, .9%–1.6%). The results were similar when evaluated at 12 months (Table 3).

### Switching to Bictegravir vs Continuing Raltegravir

Overall, 46 194 people with HIV were eligible for the target trial emulation involving switching to bictegravir vs continuing raltegravir (flowchart in Figure 1), which consisted of 672 unique individuals in the bictegravir arm and 1916 in the raltegravir arm. Median baseline CD4 cell count was similar between study arms (508 cells/mm^3^ in the bictegravir arm vs 539 cells/mm^3^ in the efavirenz arm), and most individuals were cis women. Full characteristics of included individuals are shown in Table 2, and state of residence is shown in Supplementary Table 1. No one died before the main outcome period (3 months) in the bictegravir arm (0% vs 0.06%). Outcome data were available for 28.3% in the raltegravir arm and 35.3% in the bictegravir arm.

The adjusted probability of having a viral load <50 copies/mL at 3 months was 96.9% (95% CI, 96.5%–97.3%) in those who switched to bictegravir and 95.7% (95% CI, 95.1%–96.3%) in those who continued raltegravir, which resulted in a difference of 1.2% (95% CI, .8%–1.7%). The results were similar when evaluated at 12 months (Table 3).

### Sensitivity Analyses

The results of the sensitivity analyses for all target trial emulations were generally compatible with the results from the main analyses (Supplementary Tables 2–6), as few risk differences slightly diverted from the ones of the main analyses. Of note, the sensitivity analysis based on inverse probability weighting found a risk difference of 0.8% (95% CI, −.2% to 1.2%) at 12 months in the trial emulation involving the switch to bictegravir vs the continuation of efavirenz (Supplementary Table 2). Also, the sensitivity analysis performed only among individuals with an available outcome found a risk difference of 2.3% (95% CI, −1.2% to 5.8%) at 3 months and 3.4% (95% CI, −3.1% to 8.5%) at 12 months in the trial emulation concerning the switch to bictegravir vs the continuation of raltegravir (Supplementary Table 3).

## DISCUSSION

We emulated 3 target trials using observational data from Mexico to determine the effectiveness of maintaining viral suppression when switching to bictegravir-based antiretroviral therapy as compared with continuing dolutegravir-, efavirenz-, or raltegravir-based therapy. Results suggest that those who switch from any of these 3 treatments to bictegravir are more likely to remain undetectable (viral load <50 copies/mL) at 3 months in comparison with continuing their prior treatment. Results were also very similar over 12 months of follow-up and when the definition of undetectable viral load changed to <200 copies/mL.

One prior randomized trial evaluated the comparative effectiveness of switching to bictegravir vs continuing dolutegravir (GS-US-380-184) [8]. In this noninferiority trial, approximately 560 participants who were virally suppressed were randomized to continue dolutegravir/abacavir/lamivudine or switch to bictegravir/emtricitabine/tenofovir alafenamide. Both arms achieved similar undetectability probabilities by 48 weeks (94% with bictegravir vs 95% with dolutegravir). Adverse events in the trial were mild but more common in the dolutegravir arm (8% with bictegravir vs 16% with dolutegravir), and no viral resistance mutations were found in individuals who did not maintain viral suppression. Our study estimated a higher effectiveness for bictegravir at 3 months (96% with switching to bictegravir vs 93.1% with continuing dolutegravir) and 12 months (94.3% vs 92.9%). This relatively small difference might seem of limited clinical relevance; however, one would expect about 3 additional virologic failures per 100 people who did not switch to bictegravir. Considering the context of a national treatment strategy, this gains importance. The observed difference could be due to lower adherence to treatment following adverse events or having multiple-tablet regimes in the dolutegravir arm of our study, but we do not have adverse event data to empirically confirm. Another possible explanation is that triggers to measure viral load could be biased in our study. Viral load is routinely measured following switching treatment but not always measured with the same frequency following continuation of a prior treatment. Yet, suspicion of treatment failure is an indication to measure viral load. If individuals who do not switch to bictegravir are more likely to have their viral loads measured due to suspected treatment failure, we would expect fewer of them to report undetectable viral loads. Nevertheless, the difference in likelihood of viral load measurements between those who do and do not switch would diminish as time passes, and our results are consistent when ascertained at 3 and 12 months.

Evidence on switching to bictegravir vs continuing efavirenz or raltegravir is lacking, with no evidence from randomized trials. Existing observational data include few individuals taking efavirenz- or raltegravir based-therapy or are not suited to answer questions of comparative effectiveness, as they focus on describing outcomes among those who switched to bictegravir with no comparison arm [19–24]. Thus, this study serves to fill an evidence gap, and our results suggest that switching to bictegravir could be more effective than continuing efavirenz or raltegravir.

### Limitations

Our study has several limitations. As with any observational study, we cannot rule out the presence of unmeasured or residual confounding [10]. We do not have data on clinical outcomes besides death (eg, opportunistic infections) that might influence the decision to switch therapy or on atypical causes of antiretroviral switch or continuation [25]. For example, most individuals taking raltegravir were women, which is likely due to the perceived safety of raltegravir during pregnancy as compared with other integrase strand inhibitors [2]. We also do not have data on chronic kidney disease. However, bictegravir can be used when the glomerular filtration rate is >30 mL/min or when a patient is undergoing dialysis, which means the expected proportion of ineligible people with chronic kidney disease is very low and unlikely to substantially change our conclusions [26]. Otherwise, we consider that data on the most important confounders were available and controlled for.

There were missing data on the outcome for an important percentage of individuals—for example, for the target trial that assessed switching to bictegravir vs continuing dolutegravir, there were missing outcome data for 39.2% in the “switch to bictegravir” arm and 27.6% in the “continue dolutegravir” arm. This is due to the observational nature of the data, in which persons might be followed differently according to clinical status, availability, closeness to the health care center, among other reasons. Conversely, in a switch randomized trial, study visits are strictly enforced to secure the highest percentage of outcome data, further explaining the relevant percentage of missing outcomes during the prespecified periods in our study. We assumed that these data were missing at random, conditional on baseline covariates and that predictors of missingness were the same among study arms [14–16]. While these assumptions are reasonable, the proportion of missing outcomes was nonnegligible. However, the similar results obtained when the analysis was performed only among people with an available outcome and when our outcome periods were extended support this assumption.

Our study involved a period during the COVID-19 pandemic, which may have brought changes in the follow-up of those with HIV. However, Mexico implemented considerable measures to secure the availability of antiretrovirals and follow-up to people with HIV [27].

In conclusion, our results suggest that among people with HIV and a suppressed viral load, switching to bictegravir could be more effective in maintaining viral suppression than continuing dolutegravir, efavirenz, or raltegravir.

## Supplementary Material

ofae446_Supplementary_Data

## Supplementary Data

Supplementary materials are available at *Open Forum Infectious Diseases* online. Consisting of data provided by the authors to benefit the reader, the posted materials are not copyedited and are the sole responsibility of the authors, so questions or comments should be addressed to the corresponding author.

## Notes

***Acknowledgments*.** We thank 2 anonymous reviewers for helpful feedback. I. N. thanks Sergio Iván Valdés-Ferrer for general support. We acknowledge CENSIDA staff and especially Dr Alethse de la Torre for their contribution with databases for this analysis.

***Author contributions.*** I. N. and A. A. M. designed the study and wrote the manuscript. Y. C.-V., JLM, and A. P.-M. helped with data acquisition. Y. C.-V., C. M., J. L. M., and A. P.-M. aided in methodology and investigation. I. N. performed the statistical analysis. All authors critically reviewed the manuscript and had final responsibility for the decision to submit for publication.

***Financial support***. This work was supported by the Fundación para la Salud y la Educación Dr Salvador Zubirán, A.C. (to I. N.); funds from the Strategic Research Program in Epidemiology at Karolinska Institutet, Forte (2020-00029; to A. A. M.); and the Swedish Research Council (2021-02236; to A.A.M.).
