# Supplementary material for: Comparative Effectiveness of Switching to Bictegravir From Dolutegravir-, Efavirenz-, or Raltegravir-Based Antiretroviral Therapy Among Individuals With HIV Who are Virologically Suppressed
Source: Open Forum Infect Dis. 2024 Aug 7;11(8):ofae446. doi: 10.1093/ofid/ofae446 (PMC11342391; doi:10.1093/ofid/ofae446)
Supplement: ofae446_Supplementary_Data [file ofae446_supplementary_data.docx]

**SUPPLEMENTARY MATERIAL**

**Title:** Comparative effectiveness of switching to bictegravir from dolutegravir, efavirenz, or raltegravir-based antiretroviral therapy among virally suppressed individuals with HIV **Authors:** Isaac Núñez; Yanink Caro-Vega; Conor MacDonald; Juan Luis Mosqueda; Alicia Piñeirúa-Menéndez; Anthony A. Matthews

**Content**

| Supplementary table 1. State of residence in which eligible individuals received HIV care, 2019-2021. | Page 2 |
| --- | --- |
| Description of sensitivity analyses. | Page 3-4 |
| Supplementary Table 2. Sensitivity analysis of emulated target trials using inverse probability weighting. | Page 5 |
| Supplementary Table 3. Analysis of emulated target trials among individuals with an available outcome | Page 6 |
| Supplementary Table 4. Sensitivity analysis broadening the outcome assessment period 3 months ±10 weeks and 12 months ±12 weeks. | Page 7 |
| Supplementary Table 5. Sensitivity analysis changing the definition of viral suppression to <200 copies/mL both at baseline and as the outcome. | Page 8 |
| Supplementary Table 6. Sensitivity analysis including individuals labelled as pregnant by SALVAR. | Page 9 |

**Supplementary table 1. State of residence in which eligible individuals received HIV care, 2019-2021.**

|  | **Target trial 1:**  **Switch to bictegravir vs.**  **continue dolutegravir** | | **Target trial 2:**  **Switch to bictegravir vs.**  **continue efavirenz** | | **Target trial 3:**  **Switch to bictegravir vs.**  **continue raltegravir** | |
| --- | --- | --- | --- | --- | --- | --- |
|  | Bictegravir N=5186 (%) | Dolutegravir N=364436 (%) | Bictegravir N=18073 (%) | Efavirenz N=2594984 (%) | Bictegravir  N= 673 (%) | Raltegravir  N= 45545 (%) |
| Aguascalientes | 70 (1.3) | 2217 (0.6) | 45 (0.2) | 16224 (0.6) | 3 (0.4) | 803 (1.8) |
| Baja California | 82 (1.6) | 15899 (4.4) | 816 (4.5) | 64598 (2.5) | 35 (5.2) | 2393 (5.3) |
| Baja California Sur | 35 (0.7) | 3632 (1) | 77 (0.4) | 25093 (1) | 2 (0.3) | 825 (1.8) |
| Campeche | 71 (1.4) | 750 (0.2) | 268 (1.5) | 43489 (1.7) | 9 (1.3) | 530 (1.2) |
| Chiapas | 111 (2.1) | 7007 (1.9) | 1272 (7) | 135754 (5.2) | 41 (6.1) | 2497 (5.5) |
| Chihuahua | 88 (1.7) | 7902 (2.2) | 430 (2.4) | 53055 (2) | 9 (1.3) | 448 (1) |
| Ciudad de México | 1130 (21.8) | 109990 (30.2) | 2031 (11.2) | 423211 (16.3) | 85 (12.6) | 4444 (9.8) |
| Coahuila | 17 (0.3) | 2473 (0.7) | 152 (0.8) | 12989 (0.5) | 4 (0.6) | 1616 (3.5) |
| Colima | 17 (0.3) | 2941 (0.8) | 62 (0.3) | 27801 (1.1) | 3 (0.4) | 471 (1) |
| Durango | 39 (0.8) | 2082 (0.6) | 154 (0.9) | 20642 (0.8) | 6 (0.9) | 421 (0.9) |
| Guanajuato | 197 (3.8) | 5783 (1.6) | 696 (3.9) | 84780 (3.3) | 11 (1.6) | 884 (1.9) |
| Guerrero | 106 (2) | 2517 (0.7) | 214 (1.2) | 86064 (3.3) | 13 (1.9) | 542 (1.2) |
| Hidalgo | 19 (0.4) | 2667 (0.7) | 167 (0.9) | 39892 (1.5) | 14 (2.1) | 277 (0.6) |
| Jalisco | 351 (6.8) | 31919 (8.8) | 922 (5.1) | 113514 (4.4) | 54 (8) | 3684 (8.1) |
| Michoacán | 12 (0.2) | 3453 (0.9) | 650 (3.6) | 46572 (1.8) | 4 (0.6) | 820 (1.8) |
| Morelos | 99 (1.9) | 14546 (4) | 489 (2.7) | 50864 (2) | 6 (0.9) | 465 (1) |
| México | 697 (13.4) | 62634 (17.2) | 1017 (5.6) | 276839 (10.7) | 41 (6.1) | 3705 (8.1) |
| Nayarit | 58 (1.1) | 2107 (0.6) | 319 (1.8) | 23909 (0.9) | 9 (1.3) | 2524 (5.5) |
| Nuevo León | 153 (3) | 4846 (1.3) | 1172 (6.5) | 48041 (1.9) | 23 (3.4) | 640 (1.4) |
| Oaxaca | 215 (4.1) | 3215 (0.9) | 759 (4.2) | 113447 (4.4) | 14 (2.1) | 575 (1.3) |
| Puebla | 329 (6.3) | 6061 (1.7) | 519 (2.9) | 127280 (4.9) | 41 (6.1) | 2005 (4.4) |
| Querétaro | 127 (2.4) | 3530 (1) | 402 (2.2) | 26715 (1) | 20 (3) | 778 (1.7) |
| Quintana Roo | 140 (2.7) | 18745 (5.1) | 590 (3.3) | 97290 (3.7) | 27 (4) | 2621 (5.8) |
| San Luis Potosí | 27 (0.5) | 1208 (0.3) | 121 (0.7) | 21845 (0.8) | 16 (2.4) | 946 (2.1) |
| Sinaloa | 61 (1.2) | 2762 (0.8) | 71 (0.4) | 33736 (1.3) | 5 (0.7) | 548 (1.2) |
| Sonora | 45 (0.9) | 8040 (2.2) | 136 (0.8) | 39786 (1.5) | 12 (1.8) | 556 (1.2) |
| Tabasco | 95 (1.8) | 4970 (1.4) | 969 (5.4) | 155672 (6) | 37 (5.5) | 2630 (5.8) |
| Tamaulipas | 111 (2.1) | 7538 (2.1) | 527 (2.9) | 76653 (3) | 11 (1.6) | 2792 (6.1) |
| Tlaxcala | 49 (0.9) | 4434 (1.2) | 215 (1.2) | 26501 (1) | 8 (1.2) | 598 (1.3) |
| Veracruz | 458 (8.8) | 12452 (3.4) | 1955 (10.8) | 202640 (7.8) | 76 (11.3) | 2700 (5.9) |
| Yucatán | 169 (3.3) | 5504 (1.5) | 786 (4.3) | 63842 (2.5) | 13 (1.9) | 619 (1.4) |
| Zacatecas | 8 (0.2) | 612 (0.2) | 70 (0.4) | 16246 (0.6) | 21 (3.1) | 188 (0.4) |

We performed several sensitivity analyses to test our assumptions and analytic choices. All sensitivity analyses were adjusted for the same confounders as the main analysis.

1. We performed an inverse probability (IP) weighted analysis to test our modelling assumptions and the assumption of outcome data missing at random. In it, we calculated stabilized IP weights individually for treatment assignment and for having an outcome, in both cases adjusting for the same covariates as the main analysis. Both IP weights had a mean of ~1 and were truncated at their 99 percentile. Afterwards, the product of these IP weights was used to perform a weighted binary logistic regression for the outcome. The logistic regression model was fit within the individuals with non-missing outcome data, then this model was used to standardize the risk within each arm to the distribution of baseline covariates of all eligible individuals. For computational efficiency, a random sample of 50% of the efavirenz arm was used for the emulation of target trial 2 (switching to bictegravir versus remaining on efavirenz). Large discrepancies between the inverse probability weighted analysis and the main analysis could be explained by data not missing at random conditional on the adjusted covariates.
2. We limited our analysis to individuals who had an available outcome instead of standardizing among the entire sample to test the assumption of data missing at random conditional on baseline covariates. Large discrepancies between the complete case analysis and the main analysis could be explained by data not missing at random conditional on the adjusted covariates.
3. We broadened the first outcome period to be three months ±ten weeks and the second outcome period to be twelve months ±twelve weeks to capture more outcomes. Large discrepancies between the expanded outcome period analysis and the main analysis could be explained by data not missing at random conditional on the adjusted covariates.
4. We modified the definition of undetectable viral load from <50 copies/mL to <200 copies/mL. Thus, more individuals would meet the inclusion criteria at baseline, and the outcome would be a less stringent viral load that is still considered to be undetectable viral load in HIV treatment guidelines. Similar results between study groups when using this threshold is informative when considering if switching is indicated, as someone with a viral load <200 is still considered undetectable and untransmissible.
5. We included all individuals labelled as “pregnant” to determine if there is effect modification due to possibly being misclassified as pregnant. Pregnant individuals are not eligible for a bictegravir switch, so any discrepancies with this analysis and the main analysis could be explained by misclassification.

**Supplementary Table 2. Sensitivity analysis of emulated target trials using inverse probability weighting.**

|  | **Target trial 1:**  **Switch to bictegravir vs.**  **continue dolutegravir** | | **Target trial 2:**  **Switch to bictegravir vs.**  **continue efavirenz** | | **Target trial 3:**  **Switch to bictegravir vs.**  **continue raltegravir** | |
| --- | --- | --- | --- | --- | --- | --- |
|  | **Adjusted probability, %**  **(95% CI)** | **Difference, %**  **(95% CI)** | **Adjusted probability, %**  **(95% CI)** | **Difference, %**  **(95% CI)** | **Adjusted probability, %**  **(95% CI)** | **Difference, %**  **(95% CI)** |
| **Viral load <50 copies/mL at three months** |  |  |  |  |  |  |
| Switch to bictegravir | 95.8 (94.7, 96.8) | 2.8 (1.8, 3.9) | 96.9 (96.6, 97.3) | 1.2 (0.9, 1.7) | 96.8 (96.4, 97.3) | 1.2 (0.7, 1.7) |
| Continue previous treatment | 93 (92.8, 93.2) | ref | 95.7 (95.6, 95.7) | ref | 95.7 (95.6, 95.8) | ref |
| **Viral load <50 copies/mL at twelve months** |  |  |  |  |  |  |
| Switch to bictegravir | 95.2 (93.9, 96.4) | 2.2 (0.8, 3.4) | 95.6 (94.6, 95.9) | 0.8 (-0.2, 1.2) | 95.6 (94.7, 96.5) | 0.9 (0.1, 1.9) |
| Continue previous treatment | 93.0 (92.8, 93.2) | ref | 94.7 (94.7, 94.8) | ref | 94.6 (94.5, 94.8) | ref |

CI: confidence interval. We adjusted for the following baseline covariables: CD4 cell count, living in a prison, state of residence, year of target trial, gender, and age. Further details are provided in the methods section.

**Supplementary Table 3. Analysis of emulated target trials only among individuals with an available outcome.**

|  | **Target trial 1:**  **Switch to bictegravir vs.**  **continue dolutegravir** | | **Target trial 2:**  **Switch to bictegravir vs.**  **continue efavirenz** | | **Target trial 3:**  **Switch to bictegravir vs.**  **continue raltegravir** | |
| --- | --- | --- | --- | --- | --- | --- |
|  | **Adjusted probability, %**  **(95% CI)** | **Difference, %**  **(95% CI)** | **Adjusted probability, %**  **(95% CI)** | **Difference, %**  **(95% CI)** | **Adjusted probability, %**  **(95% CI)** | **Difference, %**  **(95% CI)** |
| **Viral load <50 copies/mL at three months** |  |  |  |  |  |  |
| Switch to bictegravir | 96.1 (95.2, 96.9) | 2.8 (1.9, 3.6) | 97.1 (96.7, 97.4) | 1.3 (0.9, 1.7) | 91.1 (87.7, 94.4) | 2.3 (-1.2, 5.8) |
| Continue previous treatment | 93.3 (93.2, 93.5) | ref | 95.8 (95.7, 95.8) | ref | 88.8 (88.2, 89.3) | ref |
| **Viral load <50 copies/mL at twelve months** |  |  |  |  |  |  |
| Switch to bictegravir | 94.6 (93.4, 95.6) | 1.3 (0.1, 2.4) | 95.9 (95.4, 96.4) | 1.1 (0.5, 1.6) | 91.4 (85, 96.7) | 3.4 (-3.1, 8.5) |
| Continue previous treatment | 93.3 (93.1, 93.4) | ref | 94.8 (94.8, 94.9) | ref | 88.1 (87.4, 88.6) | ref |

CI: confidence interval. We adjusted for the following baseline covariables: CD4 cell count, living in a prison, state of residence, year of target trial, gender, and age. Further details are provided in the methods section.

**Supplementary Table 4. Sensitivity analysis broadening the outcome assessment period 3 months ±10 weeks and 12 months ±12 weeks.**

|  | **Target trial 1:**  **Switch to bictegravir vs.**  **continue dolutegravir** | | **Target trial 2:**  **Switch to bictegravir vs.**  **continue efavirenz** | | **Target trial 3:**  **Switch to bictegravir vs.**  **continue raltegravir** | |
| --- | --- | --- | --- | --- | --- | --- |
|  | **Adjusted probability, %**  **(95% CI)** | **Difference, %**  **(95% CI)** | **Adjusted probability, %**  **(95% CI)** | **Difference, %**  **(95% CI)** | **Adjusted probability, %**  **(95% CI)** | **Difference, %**  **(95% CI)** |
| **Viral load <50 copies/mL at three months** |  |  |  |  |  |  |
| Switch to bictegravir | 96.2 (95.5, 96.8) | 2.4 (1.7, 3.1) | 96.9 (96.9, 97.1) | 0.9 (0.9, 1.1) | 97 (96.6, 97.3) | 0.9 (0.6, 1.3) |
| Continue previous treatment | 93.8 (93.6, 93.9) | ref | 96.0 (96.0, 96.1) | ref | 96 (95.9, 96.1) | ref |
| **Viral load <50 copies/mL at twelve months** |  |  |  |  |  |  |
| Switch to bictegravir | 94.4 (93.3, 95.4) | 1.1 (-0.1, 2.1) | 96.0 (95.5, 96.4) | 0.9 (0.5, 1.3) | 95.9 (95.4, 96.3) | 0.9 (0.4, 1.4) |
| Continue previous treatment | 93.3 (93.2, 93.5) | ref | 95.0 (95.0, 95.1) | ref | 95 (94.9, 95.1) | ref |

CI: confidence interval. We adjusted for the following baseline covariables: CD4 cell count, living in a prison, state of residence, year of target trial, gender, and age. Further details are provided in the methods section.

**Supplementary Table 5. Sensitivity analysis changing the definition of viral suppression to <200 copies/mL both at baseline and as the outcome.**

|  | **Target trial 1:**  **Switch to bictegravir vs.**  **continue dolutegravir** | | **Target trial 2:**  **Switch to bictegravir vs.**  **continue efavirenz** | | **Target trial 3:**  **Switch to bictegravir vs.**  **continue raltegravir** | |
| --- | --- | --- | --- | --- | --- | --- |
|  | **Adjusted probability, %**  **(95% CI)** | **Difference, %**  **(95% CI)** | **Adjusted probability, %**  **(95% CI)** | **Difference, %**  **(95% CI)** | **Adjusted probability, %**  **(95% CI)** | **Difference, %**  **(95% CI)** |
| **Viral load <50 copies/mL at three months** |  |  |  |  |  |  |
| Switch to bictegravir | 98.1 (97.4, 98.8) | 2.5 (1.8, 3.2) | 98.5 (98.2, 98.8) | 1.3 (1.0, 1.6) | 98.5 (98.2, 98.7) | 1.4 (1.1, 1.7) |
| Continue previous treatment | 95.6 (95.5, 95.8) | ref | 97.2 (97.2, 97.2) | ref | 97.1 (97.0, 97.2) | ref |
| **Viral load <50 copies/mL at twelve months** |  |  |  |  |  |  |
| Switch to bictegravir | 96.9 (96.0, 97.7) | 1.5 (0.5, 2.3) | 97.9 (97.4, 98.2) | 1.3 (0.8, 1.6) | 97.8 (97.4, 98.2) | 1.2 (0.8, 1.6) |
| Continue previous treatment | 95.4 (95.3, 95.6) | ref | 96.6 (96.6, 96.6) | ref | 96.6 (96.5, 96.7) | ref |

CI: confidence interval. We adjusted for the following baseline covariables: CD4 cell count, living in a prison, state of residence, year of target trial, gender, and age. Further details are provided in the methods section.

**Supplementary Table 6. Sensitivity analysis including individuals labelled as pregnant by SALVAR.**

|  | **Target trial 1:**  **Switch to bictegravir vs.**  **continue dolutegravir** | | **Target trial 2:**  **Switch to bictegravir vs.**  **continue efavirenz** | | **Target trial 3:**  **Switch to bictegravir vs.**  **continue raltegravir** | |
| --- | --- | --- | --- | --- | --- | --- |
|  | **Adjusted probability, %**  **(95% CI)** | **Difference, %**  **(95% CI)** | **Adjusted probability, %**  **(95% CI)** | **Difference, %**  **(95% CI)** | **Adjusted probability, %**  **(95% CI)** | **Difference, %**  **(95% CI)** |
| **Viral load <50 copies/mL at three months** |  |  |  |  |  |  |
| Switch to bictegravir | 96 (95.1, 96.9) | 2.9 (2.0, 3.8) | 96.9 (96.5, 97.3) | 1.3 (0.9, 1.6) | 96.9 (96.5, 97.3) | 1.3 (0.9, 1.7) |
| Continue previous treatment | 93.1 (92.9, 93.3) | ref | 95.7 (95.6, 95.7) | ref | 95.7 (95.5, 95.8) | ref |
| **Viral load <50 copies/mL at twelve months** |  |  |  |  |  |  |
| Switch to bictegravir | 94.3 (93.1, 95.4) | 1.4 (0.1, 2.5) | 95.8 (95.3, 96.3) | 1.2 (0.7, 1.7) | 95.8 (95.2, 96.3) | 1.1 (0.5, 1.6) |
| Continue previous treatment | 92.9 (92.7, 93.1) | ref | 94.7 (94.6, 94.7) | ref | 94.7 (94.6, 94.8) | ref |

CI: confidence interval. We adjusted for the following baseline covariables: CD4 cell count, living in a prison, state of residence, year of target trial, gender, and age. Further details are provided in the methods section.
